# Severity, not type, is the main predictor of decreased quality of life in elderly women with urinary incontinence: a population-based study as part of a randomized controlled trial in primary care

**DOI:** 10.1186/1477-7525-10-153

**Published:** 2012-12-18

**Authors:** Janka A Barentsen, Els Visser, Hedwig Hofstetter, Anna M Maris, Janny H Dekker, Geertruida H de Bock

**Affiliations:** 1 Department of General Practice, University Medical Center Groningen, University of Groningen, Groningen, The Netherlands; 2 Department of Psychometrics and Statistics, Faculty of Behavioral and Social Sciences, University of Groningen, Groningen, The Netherlands; 3 Department of Epidemiology, University Medical Center Groningen, University of Groningen, Groningen, The Netherlands

**Keywords:** Population-based, Female, Urinary incontinence, Quality of life

## Abstract

**Background:**

Urinary incontinence negatively influences the lives of 25-50% of elderly women, mostly due to feelings of shame and being limited in activities and social interactions. This study explores whether differences exist between types of urinary incontinence (stress, urgency or mixed) and severity of the symptoms, with regard to their effects on generic and condition-specific quality of life.

**Methods:**

This is a cross-sectional study among participants of a randomized controlled trial in primary care. A total of 225 women (aged ≥ 55 years) completed a questionnaire (on physical/emotional impact and limitations) and were interviewed for demographic characteristics and co-morbidity. Least squares regression analyses were conducted to estimate differences between types and severity of urinary incontinence with regard to their effect on quality of life.

**Results:**

Most patients reported mixed urinary incontinence (50.7%) and a moderate severity of symptoms (48.9%). Stress urinary incontinence had a lower impact on the emotional domain of condition-specific quality of life compared with mixed urinary incontinence (r = −7.81). There were no significant associations between the types of urinary incontinence and generic quality of life. Severe symptoms affected both the generic (r = −0.10) and condition-specific (r = 17.17) quality of life.

**Conclusions:**

The effects on condition-specific quality of life domains differ slightly between the types of incontinence. The level of severity affects both generic and condition-specific quality of life, indicating that it is not the type but rather the severity of urinary incontinence that is the main predictor of decreased quality of life.

## Background

Urinary incontinence is a common condition in women. About 25% of women of reproductive age and up to 50% of postmenopausal women are troubled by this condition [1]. The International Continence Society defines urinary incontinence as ‘a complaint of any involuntary leakage of urine’ [2]. This leakage can be subdivided into three main categories: stress urinary incontinence (urinary incontinence on effort or exertion, sneezing or coughing), urgency urinary incontinence (urinary incontinence immediately preceded by urgency), and mixed urinary incontinence (both stress and urgency symptoms) [2]. Other types of incontinence, like overflow, functional, situational and nocturnal, or incontinence dependent on neurologic conditions or fistulas, are less common and are not a part of this study [2].

Whilst stress incontinence is engendered by urethral hypermobility or sphincter weakness, urgency incontinence is often caused by detrusor overactivity [3]. Besides the variation in type and cause, the levels in severity of urinary incontinence range from slight to very severe [4].

The consequences of urinary incontinence may be considerable, often causing embarrassment, stress, frustration, loss of dignity, depressive feelings and limitations in activities because of (fear of) leakage of urine [5,6]. Urinary incontinence not only has a negative effect on a woman’s physical and sexual life, but may also impede her social interactions due to insecurity about her own hygiene [3].

Because the burden of urinary incontinence differs between individuals, measurement of symptoms alone is insufficient to gain a realistic impression of this burden [7]. The burden of incontinence can depend, for instance, on the degree of acceptance and adjusting to the condition and/or on co-morbidity, the consequences of which might be considered even more burdensome [8]. A patient’s perception of her urinary incontinence, i.e. the importance that she attaches to her symptoms, also has a considerable influence on whether or not to seek help. Generally, the effect of treatment is measured by clinical observations (e.g. a change in symptoms) and by urodynamic tests. However, assessing the burden of the condition from a patient’s perspective and the impact on her quality of life is also an important part of care and treatment [9,10].

The quality of life of patients with urinary incontinence is often established with either generic instruments or with condition-specific ones. A condition-specific measurement of urinary incontinence is supposed to more specifically assess the aspects of quality of life that are impaired by urinary incontinence. Generic scales are designed to compare the effects of general health on quality of life on several (e.g. physical and social) dimensions, but may be insensitive to the influence of urinary incontinence on specific aspects of quality of life [11,12]. Thus, both these measuring instruments are associated with (the amount of) urine loss, but to a differing extent [13,14].

In addition to the variety in measuring instruments there is also diversity in study outcomes.

Studies on the effects of the three types of urinary incontinence on quality of life suggest that women with mixed and urgency urinary incontinence tend to experience a greater impact on quality of life than women with stress urinary incontinence [15,16]. The severity of incontinence is also supposed to influence the quality of life [17].

Therefore, the aim of the present study is to compare the effects of different types and levels of severity of urinary incontinence on quality of life, using both generic and condition-specific questionnaires.

## Methods

### Design

This cross-sectional study used data from the URINO project (which started in 2008) and examines the effect and cost-effectiveness of a diagnostic protocol and treatment of urinary incontinence in older women in primary care, compared to standard care [18]. The URINO project is a cluster randomized controlled trial in which the patients’ general practitioners (GPs) serve as the clusters; the patients were randomized into an intervention group and a control group.

All participants received a screening questionnaire and, if troubled by involuntary leakage at least once a month and willing to participate, were interviewed and completed a baseline questionnaire. For the present study, baseline data from the intervention group and the control group were used. Patients in the intervention group also underwent a gynecological examination and some additional tests; however, since these outcomes are irrelevant for the present study they are not further discussed here.

The URINO project was approved by the Medical Ethical Committee of the University of Groningen.

### Study population

The source population consisted of 3,684 women aged ≥ 55 years who were registered in 14 general practices in the northern part of the Netherlands. These women were known by their GP to either have or not have urinary incontinence. Of the total group, 399 women were excluded (and received no screening questionnaire) for the following reasons: currently treated for a urogynecological condition (during the previous year), had an indwelling catheter, had overflow incontinence, were suffering from malignancies, were severely demented, or were in a poor physical condition (according to their GP). The remaining women (n=3,285) received a short postal screening questionnaire on symptoms of urinary incontinence and on their willingness to participate.

The response rate was 73% (n=2,390) and, of this group, 31% (n=744) suffered from urinary incontinence (defined as involuntary leakage of urine once a month or more). Of these women with urinary incontinence, 48% (n=357) was willing to participate. Patients were then included if they were able to fill in questionnaires in Dutch and if they signed informed consent. Finally, an additional 7 women were excluded because of illiteracy or inability to complete a bladder diary, leaving a total of 350 women who provided informed consent and completed the baseline measurements.

For the present study, of the 350 available women only the first 225 participating women were included. The reason for this is that, for these 225 patients (recruited from the first general practices taking part in the main URINO study), additional detailed information on co-morbidity was available from the GPs’ registration systems.

### Measurements

Some data were collected by means of an interview and a self-administered questionnaire, whereas data on age and postal code were already known at the time of screening. After completion, participants brought their self-administered questionnaires to the center and two researchers (one per group) interviewed them, using a standardized list of questions requiring factual answers.

Besides the patient’s clinical history, the interview also covered demographic characteristics such as education level, socioeconomic status (SES) and marital status.

Both education and SES were operationalized into three levels: low, medium (called average in the case of SES) and high. The education levels are described in Table 1. For SES, participants were classified according to the postal code of the area in which they lived. These SES classes were grouped by income, employment and education (according to the Netherlands Institute for Social Research, 2006).

**Table 1:** Characteristics of the study population of female patients aged 55 years and older with urinary incontinence (N=225) N (%) or mean SD

Age (years)		65.4 9.57
Body mass index		27.6 5.2
Charlson index a	Value 0 - 1	193 (55.4)
	Value 2 - 3	30 (8.6)
	Value 6	2 (0.6)
Education b	Low	59 (26.2)
	Medium	101 (44.9)
	High	64 (28.4)
	Unknown	1 (0.4)
Socioeconomic status score c	Low	44 (19.6)
	Average	167 (74.2)
	High	14 (6.2)
Marital status	No partner	56 (24.9)
	Partner	115 (51.1)
	Unknown	54 (24.0)
Self-reported type of urinary incontinence	Stress incontinence	64 (28.4)
	Urgency incontinence	40 (17.8)
	Mixed incontinence	114 (50.7)
	Unknown	7 (3.1)
Severity of urinary incontinence according to the Incontinence Severity Index	Slight	59 (26.2)
	Moderate	110 (48.9)
	Severe	43 (19.1)
	Very severe	8 (3.6)
	Unknown	5 (2.2)

^a^ Charlson index: weighted risk of mortality < 1 year, dependent on co-morbidity (score 6 as the highest risk).

^b^ Low: primary/junior secondary vocational education; medium: secondary/senior vocational/higher secondary education; high: higher professional/university education.

^c^ Dependent on income, employment and educational level according to the postal code of the area the participants live in (Netherlands Institute for Social Research, 2006).

The weight and height of participants were measured to calculate body mass index (BMI).

The self-administered questionnaires comprised questions on the frequency and amount of involuntary loss of urine, assessed by the Incontinence Severity Index (ISI) [4]; this is measured on a 12-point scale by multiplying the frequency of losing urine (once a month, couple of times monthly, couple of times weekly or every day or night) and severity (losing drops, puddles or more). Further information on the symptoms of urinary incontinence was measured by the Urinary Distress Inventory (UDI) and the International Consultation Incontinence Questionnaire-Urinary Incontinence Short Form (ICIQ-UI-SF) [19]. The answers to the questionnaires were used to establish the (self-reported) type of urinary incontinence.

Stress urinary incontinence was assumed to be present when patients gave one of the following answers to the question ‘When do you lose urine?’ from the ICIQ-UI-SF: ‘*I lose urine during physical activity*’ or ‘*I lose urine when I cough or sneeze*’. Women who answered this same question with ‘*I lose urine before I can reach the toilet*’ or who confirmed one question from the UDI, i.e. that they lost urine with the urgency to urinate (‘Do you have involuntary leakage of urine when you feel the urgency to urinate?’) were assumed to suffer from urgency incontinence. Both symptoms refer to mixed urinary incontinence.

The generic quality of life was assessed by a questionnaire on health outcome and utilities, i.e. the Euroqol 5D (EQ-5D). The EQ-5D is designed to complement other quality of life measurements (such as condition-specific ones) and consists of five questions on different aspects of health: mobility, self-care, usual activities, pain, and psychological status. The score depends on the person’s own judgment regarding health status and ranges from −0.33 (serious problems with the mentioned aspects) to 1 (no problems at all) [20,21].

The condition-specific quality of life, similar to the influence of urinary incontinence on (social) activities and wellbeing, was measured with the Incontinence Impact Questionnaire (IIQ-7). Scores on the IIQ-7 range from 0–100, where 0 indicates no impact and 100 indicates very high impact of urinary incontinence on four domains, i.e. physical activity, social relationships, travelling and emotional health [22]. Scores on the four sub-domains are obtained on an ordinal range (0, 16.67, 33.33, 50.00, 66.67, 83.33 and 100) and their sum is the IIQ-7 total score (range 0–400).

Data on co-morbidity were withdrawn from the registration systems of the GPs, according to the Charlson index, which predicts the one-year mortality for patients with a range of co-morbid conditions (taking into account both the number and severity of conditions) [23-25].

### Analysis

Primary outcome of this study was the condition-specific quality of life, as measured by the IIQ-7 and the secondary outcome was the generic quality of life as defined by the score on the EQ-5D. Determinants were the self-reported types of urinary incontinence and the severity of the symptoms, measured by the ICIQ-UI-SF, and by one question from the UDI and the ISI, respectively.

First, the patient characteristics at baseline (age, BMI, SES, marital status, education) and co-morbidity were described, as well as the self-reported type and severity of urinary incontinence. Next, correlations were calculated between all possible confounding variables as mentioned above and the outcome variables (the EQ-5D and IIQ-7), using Spearman’s correlation test. A high score on the EQ-5D indicates a higher quality of life, whereas a high score on the IIQ-7 indicates a large impact and therefore a diminished quality of life.

Finally, associations between the types and severity of urinary incontinence and the outcome variables were assessed. The categories severe and very severe urinary incontinence were taken together due to the small number of patients reporting very severe symptoms. Multilevel analysis was performed to take into account dependency in the data between patients with the same GP.

The dependent structure between patients and GPs as measured by the residual ICC was low (ρ=0.004), therefore an ordinary least squares (OLS) regression analysis was performed. Regarding the outcome variable EQ-5D, no indication for violating the assumptions of the OLS regression analysis was found. However, violation of the normality assumption was found for the IIQ-7. As ordinal and logistic regression analysis yielded the same conclusions, only the results of the OLS regression analyses are reported.

Scores on the IIQ-7 were missing for 14 patients. Furthermore, an additional 49 patients had a missing score on marital status. Five other patients had missing data for type of urinary incontinence and two other patients had missing data for severity of urinary incontinence; this resulted in a total of 155 patients available for analysis of the IIQ-7 scores.

For EQ-5D, the score was not available for 6 patients. Also, for an additional 51 patients no data were available on marital status. Five other patients had missing data for type of urinary incontinence and another patient had a missing value for severity of urinary incontinence; this resulted in a total of 162 patients available for analysis of the EQ-5D.

All data were analyzed using SPSS version 17.

## Results

The baseline characteristics of the study population and their self-reported types and severity of urinary incontinence are presented in Table 1. Of the 225 respondents, the minimum age at the moment of screening was 55 years, mean age was 65.4 (SD=9.57) years.

Whereas the majority reported mixed urinary incontinence (50.7%), the participants were also troubled with stress (28.4%) and urgency (17.8%) incontinence. Regarding the degree of severity of complaints, for 48.9% it was moderate, for 26.2% it was slight, for 19.1% it was severe, and 3.6% of the women reported very severe complaints.

Table 2 shows that age is weakly and negatively related to the primary outcome EQ-5D. BMI also has a weak negative correlation with the EQ-5D, but is weakly and positively related to the IIQ social domain. SES correlates weakly and negatively with the IIQ physical activity and the emotional health domain, as well as with the total IIQ-7 score. There is a moderate and negative correlation between co-morbidity and the EQ-5D, and education is weakly and negatively associated with both the IIQ physical and social domain, whereas marital status has a weak and positive relationship with the IIQ social domain.

**Table 2:** Spearman’s correlations between patient characteristics and quality of life

	**Age**	**Body mass index**	**SES**^**a**^	**Marital status**	**Education**	**Charlson index**^**b**^
EQ-5D^c^	−0.17*	−0.20**	0.09	−0.11	0.08	−0.31**
IIQ ^d^ Physical activity	0.10	0.03	−0.16*	0.09	−0.20**	−0.05
IIQ Social relationships	0.05	0.14*	−0.09	0.15*	−0.16*	0.05
IIQ Travelling	−0.01	−0.03	−0.10	0.06	−0.12	−0.06
IIQ Emotional health	0.08	−0.03	−0.17*	0.11	−0.02	0.02
IIQ Total score	0.05	0.02	−0.15*	0.12	−0.11	−0.06

* Correlation is significant at the 0.05 level (2-tailed). ** Correlation is significant at the 0.01 level (2-tailed).

^a^ Socioeconomic Status (SES); Dependent on income, employment and educational level according to the postal code of the area the participants live in (Netherlands Institute for Social Research, 2006).

^b^ Charlson Index: weighted risk of mortality <1 year, dependent on co-morbidity.

^c^ Euroqol 5D (EQ-5D); range −0.33 to 1 with lower numbers indicating a greater decrease in quality of life.

^d^ Incontinence Impact Questionnaire (IIQ); range 0 to 100 with higher numbers indicating a greater decrease in quality of life.

As shown in Table 3, after adjusting for the effect of confounders, patients with severe urinary incontinence score significantly lower on the EQ-5D and significantly higher on the IIQ-7 total score, and on all sub-domains, compared to patients with slight severity of urine loss. Furthermore, also after adjusting for the effect of confounders, patients with stress urinary incontinence score significantly lower on the emotional health domain compared to patients with mixed urinary incontinence, whilst there is no difference between urgency urinary incontinence and mixed urinary incontinence.

**Table 3:** Results of ordinary least square regression analyses for general and condition-specific quality of life measurements

	**EQ-5D**^**d**^**(N=162)**	**IIQ**^**e**^**total (N=155)**	**IIQ phys (N=155)**	**IIQ travel (N=155)**	**IIQ social (N=155)**	**IIQ emot (N=155)**
	**Score −0.33- 1**	**Score 0 - 400**	**Score 0 - 100**	**Score 0 - 100**	**Score 0 - 100**	**Score 0 - 100**
*Coefficients*^*a*^						
SES	0.16*	−0.15	−0.18*	−0.10	−0.09	−0.17*
Marital status	−0.01	3.31	1.40	1.26	7.33	4.09
Age	0.03	−0.20*	−0.10	−0.17	−0.12	−0.16
BMI	−0.05	−0.05	−0.01	0.01	0.09	−0.09
Education	−0.11	−0.02	−0.16	−0.01	−0.01	0.02
Co-morbidity	−0.26**	−0.02	−0.09	−0.03	0.01	0.08
R2	0.12**	0.06	0.10**	0.03	0.06	0.07
*Type of urinary incontinence* (*UI*) ^b^						
Stress	0.01	−4.15	−3.90	−4.62	−1.90	−7.81*
Urgency	−0.06	2.38	−3.34	3.38	−1.61	0.98
R2 change	0.02	0.04*	0.02	0.04	0.01	0.06**
*Severity of UI*^c^						
Moderate	0.00	1.57	2.19	1.98	1.25	2.15
Severe	−0.10**	17.17**	14.87**	13.89**	22.92**	21.72**
R2 change	0.06**	0.15**	0.09**	0.07**	0.12**	0.14**

*p < 0.05. **p < 0.01.

^a^ Standardized coefficients noted for SES, Age, BMI, Education, and Co-morbidity. Unstandardized coefficients noted for Marital status, Type UI, and Severity UI.

^b^ Reference mixed UI.

^c^ Reference slight UI.

^d^ Euroqol 5D (EQ-5D); range from −0.33 to 1 with lower numbers indicating a greater decrease in quality of life.

## Discussion

The present study assessed the impact of different levels of severity and types of urinary incontinence on the patient’s generic and condition-specific quality of life.

Some characteristics of our study population were also significantly related to the quality of life and (the most relevant) are described in the Results section. In summary: a higher age, higher BMI and more co-morbidity negatively influences the generic quality of life. The negative relation between SES and the condition-specific quality of life indicates the opposite, because the higher the SES the better the quality of life.

However, the most important outcome is that about half of the participants were troubled by mixed urinary incontinence (50.7%) and a moderate severity of urine loss (48.9%); this mixed urinary incontinence had a greater impact on the emotional domain of the condition-specific quality of life as compared to stress urinary incontinence, although there is no difference compared with urgency urinary incontinence.

Women with symptoms of stress urinary incontinence are less dependent on their urine loss because they tend to lose urine during situations which are generally known to them and thus might be avoided [26]. However, the degree of severity of the incontinence influences both the generic and the condition-specific quality of life.

Compared to patients with slight urinary incontinence, patients with severe urinary incontinence (23%) experience more impact of urinary incontinence on all domains of the condition-specific quality of life, as well as on the condition-specific quality of life total score. Moreover, severe urinary incontinence also diminishes the generic quality of life compared to slight urinary incontinence, whilst there is no effect of moderate severity on quality of life as compared to slight severity of urine loss. Therefore, it appears that the severity of the symptoms, rather than the type of urinary incontinence, is a greater predictor for a decreased quality of life.

### Comparison with existing literature

As mentioned above, mixed urinary incontinence is associated with lowered condition-specific quality of life as compared to stress urinary incontinence, as also reported by Schimf et al. [16], whereas there is no significant difference with regard to urgency urinary incontinence, as also reported by Frick et al. [15].

When increased, symptom severity (as measured by the IIQ-7) diminishes the condition-specific quality of life in general, as also found by Tennstedt et al. [6] and Huang et al. [27].

No differences were found between the types of urinary incontinence and their effect on the generic quality of life. This result is consistent with findings of Grimby et al. [28] and Coyne et al. [26] but in contrast to Botlero et al. [29] who argue that mixed urinary incontinence is associated with a larger reduction in overall wellbeing. However, in the latter study mixed urinary incontinence had more impact on emotional wellbeing and mood, which in our study is described as the emotional domain of the condition-specific quality of life. Differences in the choice of measurements and interpretation might be the reasons for the differences found between these studies.

### Strengths and limitations

In this population-based study, women were included on the basis of randomizing their GP. However, because these women are a random selection (based on age ≥ 55 years), at the time of screening it is unknown whether they have already asked for help, either recently or in the past.

In the primary care setting these women with urinary incontinence have rarely been studied.

In the present study, different types of urinary incontinence as well as the severity of its symptoms and effects on quality of life were explored, whereas other studies mainly investigated either the type or the severity of urinary incontinence.

Also, this study uses both generic and condition-specific questionnaires, which is recommended by Dugan et al. [13] and Naughton et al. [21]; the latter in particular report that the IIQ and EQ-5D are highly recommended. The use of both questionnaires allows to assess symptom distress as well as general wellbeing.

Self-administered questionnaires (allowing a person to judge their own health status) are important for the present study. However, self-reports on the type of urinary incontinence and severity of symptoms can lead to a potential bias in data because of subjective symptom perception [3].

Another potential limitation of this study is the inclusion of women who participated because they already have symptoms, thus precluding comparison with women without urinary incontinence. Also, women suffering from urinary incontinence may already experience a somewhat diminished quality of life.

The willingness to participate was 48%, which was more than expected at the start of the trial. However, any loss is a limitation and in this study the main reasons for non-response were: too great a burden to participate (19.7%), not wishing to undergo more examinations/research in this or other areas (17.4%), and being too old (15.5%, with a mean age of 72 years).

Also, because this study has a cross-sectional design with only one measuring point, the direction of the causal relationship between urinary incontinence and (factors of) quality of life cannot be determined, as this association could also be bidirectional.

Finally, because this study included only women in the Netherlands aged ≥ 55 years, these results cannot be extrapolated to other cultures and are not generalizable to all ages.

### Implications for future research

The use of different types of measurements can provide valuable information for future research. However, we need to establish which measuring instrument is most effective to study the relation between urinary incontinence and quality of life, or whether the use of two or more questionnaires may be a better approach to obtain maximum insight on this topic [11,13].

We recommend to perform a longitudinal study, with several measuring points, to assess whether changes in symptoms are related to changes in quality of life. Performing a population-based study will allow comparisons to be made between patients with and without urinary incontinence, and to establish differences in generic quality of life due to the impact of urinary incontinence.

More research is required on differences between the types urinary incontinence and their effect on quality of life, especially with regard to urgency urinary incontinence.

In addition, the level of burdensomeness may differ between age groups because some symptoms and complaints are often assumed (like co-morbidity) to ‘belong’ to older age. Studying younger women with urinary incontinence (albeit a smaller group) may increase our knowledge on the impact of urinary incontinence on the quality of life.

Finally, due to the aging of society and knowing that urinary incontinence increases with age, early detection and treatment is an important part of future care and other considerations related to rising costs.

## Conclusions

This study shows that the severity of symptoms, not the type of urinary incontinence, is a greater predictor for a decreased (generic and condition-specific) quality of life. Knowledge on the impact of the level of severity, in relation to the burden for incontinent women, may increase GPs’ insight into the consequences of the symptoms of urinary incontinence, with the aim to improve care and increase the patient’s quality of life.

## Abbreviations

BMI: Body Mass Index; EQ-5D: Euro Qol 5 Dimensions; SES: Socioeconomic status; IIQ-7: Incontinence Impact Questionnaire – short form.

## Competing interests

All authors declare they have no conflicts of interest.

## Authors' contributions

EV, JHD and GHB were responsible for study design and conceptualization, as well as the drafting and revising (besides JAB) of the manuscript. JAB and EV collected and interpreted the data and AMM, HH and JAB performed the statistical analysis. All authors have read and approved the final manuscript.
